# An Update on Wastewater Multi-Resistant Bacteria: Identification of Clinical Pathogens Such as *Escherichia coli* O25b:H4-B2-ST131-Producing CTX-M-15 ESBL and KPC-3 Carbapenemase-Producing *Klebsiella oxytoca*

**DOI:** 10.3390/microorganisms9030576

**Published:** 2021-03-11

**Authors:** Elsa Mesquita, Rita Ribeiro, Carla J. C. Silva, Rita Alves, Rita Baptista, Sílvia Condinho, Maria João Rosa, João Perdigão, Cátia Caneiras, Aida Duarte

**Affiliations:** 1 Water Quality and Treatment Laboratory, Urban Water Unit, Hydraulics and Environment Department, The Laboratório Nacional de Engenharia Civil (LNEC)—National Civil Engineering Laboratory, 1700-066 Lisboa, Portugal; emesquita@lnec.pt (E.M.); rribeiro@lnec.pt (R.R.); mjrosa@lnec.pt (M.J.R.); 2 Direção de Operações de Saneamento, ETAR de Beirolas, Rua Chen He, 1990-513 Sacavém, Portugal; carla.silva@adp.pt (C.J.C.S.); rita.alves@adp.pt (R.A.); 3 Laboratório do Sotavento da Águas do Algarve, Rua do Repouso, n° 10, 8000-302 Faro, Portugal; r.baptista@adp.pt (R.B.); s.condinho@adp.pt (S.C.); 4 Research Institute for Medicines (iMed.ULisboa), Faculty of Pharmacy, Universidade Lisboa, Av. Gama Pinto, 1649-003 Lisboa, Portugal; jperdigao@ff.ulisboa.pt; 5 Microbiology Research Laboratory of Environmental Health (EnviHealthMicro Lab), Institute of Environmental Health (ISAMB), Faculty of Medicine, Universidade de Lisboa, 1649-028 Lisboa, Portugal; ccaneiras@medicina.ulisboa.pt; 6 Institute of Preventive Medicine and Public Health (IMP&SP), Faculty of Medicine, Universidade de Lisboa, 1649-028 Lisboa, Portugal; 7 Department of Microbiology and Immunology, Faculty of Pharmacy, Av. Gama Pinto, 1649-003 Lisboa, Portugal; 8 Centro de Investigação Interdisciplinar Egas Moniz, Instituto Universitário Egas Moniz, 2829-511 Monte da Caparica, Portugal

**Keywords:** wastewater treatment plant (WWTP), CTX-M-15, SED-1, ESBL, KPC-3 carbapenemase, *Klebsiella oxytoca*, *Ochromobactrum intermedium*, *Citrobacter farmeri*, Portugal

## Abstract

Wastewater treatment plants (WWTPs) are significant reservoirs of bacterial resistance. This work aims to identify the determinants of resistance produced by Gram-negative bacteria in the influent and effluent of two WWTPs in Portugal. A total of 96 wastewater samples were obtained between 2016 and 2019. The numbers of total aerobic and fecal contamination bacteria were evaluated, and genomic features were searched by polymerase chain reaction (PCR) and Next-Generation Sequencing (NGS). *Enterobacteriaceae* corresponded to 78.6% (*n* = 161) of the 205 isolates identified by 16sRNA. The most frequent isolates were *Escherichia* spp. (57.1%, *n* = 117), followed by *Aeromonas* spp. (16.1%, *n* = 33) and *Klebsiella* spp. (12.7%, *n* = 26). The remaining 29 isolates (14.1%) were distributed across 10 different genera. Among the 183 resistant genes detected, 54 isolates produced extended spectrum β-lactamases (ESBL), of which *bla*_CTX-M-15_ was predominant (37 isolates; 68.5%). A KPC-3 carbapenemase-producing *K. oxytoca* was identified (*n* = 1), with *bla*_KPC-3_ included in a transposon *Tn4401* isoform b. A higher number of virulence genes (VG) (19 genes) was found in the *E. coli* 5301 (O25b-ST131-B2) isolate compared with a commensal *E. coli* 5281 (O25b-ST410-A) (six genes). Both shared five VG [Enterobactin; Aerobactin, CFA/1 (clade α); Type1 (clade γ1); Type IV]. In conclusion, this work highlights the role of relevant clinical bacteria in WWTPs, such as KPC-3-producing *K. oxytoca,* and, for the first time, a CTX-M-15-producing *Ochromobactrum intermedium*, a human opportunistic pathogen, and a SED-1-producing *Citrobacter farmeri*, an uncommon CTX-M-type extended-spectrum beta-lactamase.

## 1. Introduction

Resistance to antibiotics is a natural biological phenomenon due to evolution by natural selection. According to Sanderson et al. 2016 [1], antibiotic resistance genes (ARGs) should be considered a biological contaminant of emerging concern, considering that they are able to transfer, through genetic processes, to multiple types of organisms and over long distances. Increasing resistance to antibiotics has led to the investigation of different origins, such as wastewater treatment plants (WWTPs), which have long been considered to be reservoirs of bacterial resistance [2,3]. Although multidrug-resistant (MDR) bacteria are ubiquitous in the environment, WWTPs are a key source of antibiotic resistance determinants outside hospitals, potentially providing a selective pressure for multidrug resistance [4,5]. WWTPs have dual functions, as they can serve either as hotspots for gene transfer between bacteria due to their high-nutrient and high-density load [6], or as reservoirs of ARGs, acting as repositories or facilitating the long-term storage of antibiotic resistance determinants [1]. In fact, WWTPs may accelerate the evolutionary timeline of ARGs by increasing the mobilization of environmental resistance genes into pathogenic bacteria [7].

WWTPs generally have three stages: primary, secondary, and tertiary treatment. During these processes, considerable changes occur in the distribution of the bacterial population, but the general observation in the literature is that treatment leads to a significant reduction in bacterial numbers, including the total numbers of resistant bacteria [8]. There are two main approaches to determine the occurrence of MDR or ARGs in wastewater: culture or molecular based, each with specific advantages [6]. Whole-genome sequencing (WGS) is revolutionizing genomic medicine and public health, and has significantly improved our ability to analyze outbreak scenarios, characterize the transmission dynamics of important pathogens, and assess horizontal transfer mediated by resistance-mobilizing genetic elements [9]. Conventional microbiology, as well as molecular biology methods, can be complementary for the presence/absence analyses of pathogens, and their genetic targets of antibiotic resistances.

The aim of this study was to monitor the occurrence of multiresistant bacteria, and characterize the antibiotic resistance genes of isolates from wastewaters from the Beirolas-Lisboa North and Faro Northwest WWTPs in Portugal, including an overview on the role of WWTPs as a barrier against MDR bacteria.

## 2. Materials and Methods

### 2.1. Phenotypic Characterization of Isolates from Wastewater

#### 2.1.1. Sample Sites and Collection

Influent and effluent samples from two WWTPs, Beirolas-Lisboa North and Faro Northwest in Algarve, Portugal, were randomly collected on a monthly basis from November 2016 to January 2019. A total of 96 wastewater samples were collected in sterile bottles (1 L), transported in refrigerated conditions to the Bacteriology Laboratory of the Microbiology and Immunology Department, Faculty of Pharmacy, University of Lisboa, and were processed on the same day.

#### 2.1.2. Microbiological Analyses

Microbiological analyses were performed using standardized methods according to ISO 7704 (evaluation of membrane filters used for microbiological analyses) [10]; ISO 8199 (water quality-general requirements for colony count by inoculation in a solid medium) [11]; and ISO 9308-1 (enumeration of *Escherichia coli* and coliform bacteria—Part 1: membrane filtration method for water) [12]. Briefly, 1 mL of each wastewater sample collected was taken, and serial dilutions were made in sterile peptone water until a dilution of 10^7^ was reached. The dilutions for influent wastewater samples ranged from 10^4^ to 10^7^, and effluent samples ranged from 10^1^ to 10^4^. One mL (1 mL) of each dilution was filtered through a 0.45 μm pore-size sterile membrane filter (Millipore Co, PVL, Famões, Portugal). The membrane filters were then placed into specific culture media: (i) COMPASS^®^ cc Agar (Biokar Diagnostics, PVL, Famões, Portugal) which allows the direct enumeration of *Escherichia coli* and coliforms, in 24 h; (ii) COMPASS^®^ *Enterococcus* Agar (Biokar Diagnostics, PVL, Famões, Portugal), a selective media used for the enumeration of enterococci in food and water; or (iii) Plate Count Agar (PCA) to enumerate aerobic bacteria containing glucose and yeast extract (Biokar Diagnostics, PVL, Famões, Portugal). Membrane filtration is a reliable and recommended method that allows the use of large sample volumes. However, it is more suitable for the counting of specific bacteria deposited on the filter membrane in selective media, which inhibits the growth of other bacteria, making it easier to count the colonies. It is not a suitable method for enumerating total aerobic bacteria in waters with high bacterial background flora, because is not easy to count the colonies, which cannot be perfectly separated, and a reproducible count is not allowed. Therefore, in parallel with a filtration membrane, 0.1 mL of each dilution of influent and effluent wastewater was spread on the PCA medium surface with disposable L-shaped cell spreaders (VWR International, Amadora, Portugal). The plates were incubated under aerobic conditions at 37 °C, and growth was observed after 24 h, for total aerobic bacteria and coliforms, and at 44 °C for 24 h for *E. coli* and *Enterococcus* spp. Bacterial counts were expressed as colony-forming units per mL (CFU/mL), and were considered the total CFUs per membrane or plate within the range of 15–300 or 1–100, respectively. For each specific culture medium, only the plates within the range of 1–100 typical colonies were considered for the bacterial count.

#### 2.1.3. Isolation of Resistant Gram-Negative Bacteria

Antibiotics were individually added into Drigalsky Lactose agar (DLA) medium to obtain counts of antibiotic resistant Gram-negative bacteria. The DLA culture medium was used for the selection of resistant bacteria, as it allows the differentiation of bacteria according to their ability to ferment or not the lactose, which is revealed by difference of color (yellow or blue, respectively). The medium was prepared with the incorporation of new antibiotic solutions to give final concentrations as follows: cefotaxime (β-lactam) 2 mg/L; gentamicin (aminoglycosides) 25 mg/L; norfloxacin (fluoroquinolone) 4 mg/L; and sulfamethoxazole 256 mg/L, according to the international standard ISO 20776-1:2006 revised by ISO 20776-1:2019. The selection of these antibiotics was made according to the clinical relevance to human health.

A total of 0.1 mL of each dilution 10^2^ (influent) and 10^1^ (effluent) were spread on the DLA surface, with and without antibiotics. Different dilutions were used for the influent and effluent as influent is untreated wastewater and effluent is treated wastewater. Then, the growth was observed after 24 h of incubation at 37 °C, and the lactose-positive or -negative colonies were counted. The results obtained with and without antibiotic were compared, in order to quantify and determine the average CFU per mL and, consequently, the resistance of isolates to these antibiotics. For pure cultures, colonies showing different morphologies were selected and transferred to new DLA medium with selective antibiotics (presented above). After incubation, the isolates were conserved at −80 °C in stock medium (BHI broth supplemented with 20% glycerol) for further phenotype and genotype characterization.

The effectiveness of wastewater treatment processes was measured using a concept called “log removal values” (LRVs). The LRV is a measure of the ability of a treatment process to remove pathogenic microorganisms. LRVs are determined by taking the logarithm of the ratio of the pathogen concentration in the influent and effluent water of a treatment process, according to the following equation:

$$\mathrm{LRV}=\log_{10}\left\{\frac{\mathrm{Influent}\;\mathrm{Pathogen}\;\mathrm{Concentration}}{\mathrm{Effluent}\;\mathrm{Pathogen}\;\mathrm{Concentration}}\right\}$$

(i) an LRV of 1 is equivalent to 90% removal of a target pathogen; (ii) an LRV of 2 is equivalent to 99% removal; (iii) an LRV of 3 is equivalent to 99.9% removal [13].

#### 2.1.4. Antibiotic Susceptibility Testing

Antibiotic susceptibility phenotypes were determined by the agar diffusion technique based on the Kirby–Bauer method, according to standard recommendations of the European Committee on Antimicrobial Susceptibility Testing (EUCAST, Disk Diffusion Test Methodology, 2017), with the following antibiotic disks: cefoxitin (FOX) 30 μg; cefotaxime (CTX) 5 μg; ceftazidime (CAZ) 10 μg; imipenem (IPM) 10 μg; gentamicin (GM) 10 μg; ciprofloxacin (CIP) 5 μg; and trimethoprim/sulfamethoxazole (SXT) 1.25 μg/23.75 μg (Bio-Rad, Portugal). Bacterial suspensions, with turbidity to 0.5 MacFarland Standard were spread on Mueller Hinton agar (Biokar Diagnostics, PVL, Famões, Portugal) using a sterile cotton swab. After 24 h of incubation at 35 °C, the diameters of antibiotic inhibition of growth were measured, and interpretation of zone diameters was undertaken according to EUCAST breakpoints, 2018. An isolate is considered multiple drug resistant (MDR) if it is resistant to three or more antibiotic classes [14].

### 2.2. Genomic Characterization of Isolates from Wastewater

#### 2.2.1. DNA Extraction and Sequencing

DNA was extracted from pure isolates using the NZY Microbial gDNA Isolation Kit (NZYTech, Lisboa, Portugal), following the manufacturer’s protocol. Certain steps in the protocol were modified to obtain better DNA yield, which included increasing the bead-lysis step for certain cultures from 10 min to 20 min. Furthermore, the elution step incubation period was increased by an additional 5 min. The concentration and purity of DNA was determined via a nanophotometer (NanoDrop^®^, Isogen Life Science, De Meern, The Netherlands). The extracted DNA was stored at −20 °C until it was used for polymerase chain reactions (PCR) and sequencing by Sanger and Next-Generation Sequencing (NGS) technology (Stab Vida, Lisboa, Portugal).

#### 2.2.2. Genotype Identification of Isolates

The identification of resistant isolates was completed by 16S rRNA gene sequencing. This methodology can identify bacteria that have not been identified using manual and automated systems, and can characterize previously undescribed species. Universal 16S rRNA bacterial primers 27F (5′-AGAGTTTGATCCTGGCTCAG-3′) and 1392R (5′-GGTTACCTTGTTACGACTT-3′) were used to amplify this gene using 10 ng of genomic DNA from each strain. PCR products were visualized on a 1% agarose gel stained with Gel Red (NZYTech, Lisboa, Portugal) under UV light, to confirm the presence of a 1350 base pairs (bp) band.

#### 2.2.3. Detection of Antibiotic Resistance Genes

According to the antibiotype profiles, the isolates suspected of producing β-lactamases and carbapenemases were screened for *bla*_CTX-M-_, *bla*_KPC-_, and *bla*_OXA-_ genes. The presence of Class1 integrons and the plasmid-mediated quinolone resistance genes *aac(6′)-Ib-cr*, *qnrA*, *qnrB*, and *qnrS* were characterized using primers and conditions previously described [15,16,17]. The primers used to amplify the *bla*_OXA_- genes were 5′-GTACTAATCAAAGTGTGAA-3′ and 5′-TTCCCCTAACATGAATTTGT-3′. Studies of the genetic structure surrounding the *bla*_KPC-3_ gene, in order to identify the Tn3-based transposon, *Tn4401*, were undertaken as previously described [3].

#### 2.2.4. Phylogenetic Group, Molecular Typing, and Sequence Analysis

For identification of phylogenetic group associations in *E. coli*, a triplex PCR method was employed using primers targeting two genes, *chuA* and *yjaA*, and one anonymous DNA fragment, TspE4C [18]. Allele-specific PCR was performed to identify the O25-ST131 clone of *E. coli*. Primers O25pabBF (5′-TCCAGCAGGTGCTGGATCGT-3′) and O25pabB.R (50-GCGAAATTTTTCGCCGTACTGT-3′) were used to amplify a 347 bp fragment of the *pabB* gene specifically in isolates belonging to the O25b-ST131 clone. A positive PCR control was included in the assay to confirm that any amplification failure with the *pabB* allele-specific primers was not due to poor DNA quality or to failure of the PCR itself. This control PCR targeted a 427 bp fragment of the *trpA* gene, amplified with primers trpA.F (5′-GCTACGAATCTCTGTTTGCC-3′) and trpA2.R (5′-GCAACGCGGCCTGGCGGAAG--3′) [19].

Multilocus sequence typing (MLST) typing was performed, in a representative number of *E. coli* strains, by PCR amplification and sequencing of seven housekeeping genes (*adk*, *fumC*, *gyrB*, *icd*, *mdh*, *purA* and *recA*), following the protocols specified on the *E. coli* MLST website, as well as the primer sequences of seven genes available at http://mlst.warwick.ac.uk/mlst/dbs/Ecoli (accessed on 15 December 2020). Allele numbers for seven gene fragments of each isolate were obtained by comparing with corresponding alleles, and the Sequence Type (ST) of each isolate was determined by combining seven allelic profiles in the MLST *E. coli* database (http://mlst.warwick.ac.uk/mlst/dbs/Ecoli, accessed on 15 December 2020). All amplification products were purified by NZYGelpure Kit (NZYTech, Lisboa, Portugal), following the manufacturer’s protocol. DNA fragments of different sizes were sequenced by STAB VIDA (https://www.stabvida.com/, accessed on 15 December 2020). The nucleotide sequences were analyzed using the NCBI Nucleotide-BLAST database (https://blast.ncbi.nlm.nih.gov/Blast.cgi, accessed on 15 December 2020), and to generate alignments between three or more sequences we used the multiple sequence alignment program Clustal Omega (https://www.ebi.ac.uk/Tools/msa/clustalo/, accessed on 15 December 2020).

#### 2.2.5. Whole-Genome Sequencing and Sequence Analysis

Two *E. coli* 5301 (O25b:H4-B2-ST131) and *E. coli* 5281 (O25b:H4-A-ST410) were selected for whole-genome sequencing. A total of 1 μg DNA, prepared according to the description in Section 2.1, was sent to STAB VIDA (http://www.stabvida.com/, accessed on 15 December 2020) for genome sequencing on the Illumina Platform PE150 with ~100–150× of coverage. The de novo assembly approach was used, which utilizes an algorithm based on de Bruijn graphs [20]. After the initial contig creation, the reads were mapped back to the contigs for assembly correction. The genome assemblies were evaluated with QUAST 5.0.2. The assembly statistics and cumulative plots were created [21]. After obtaining the assemblies, an identification and characterization of the bacteria was performed using the services of the Center for Genomic Epidemiology (CGE). The identification of acquired resistance genes used ResFinder 4.0 [22], and virulence genes used VirulenceFinder 2.0. [23]. Additionally, virulence genes were detected by aligning them directly against the Virulence Factors of Pathogenic Bacteria Database (VFDB; “core dataset” downloaded on 12 December 2020) [24]. PlasmidFinder v2.1 [25] was used to detect the incompatibility groups of plasmids, and SerotypeFinder2.0 confirmed the previously determined serotype [26].

## 3. Results

### 3.1. Phenotypic Characterization

#### 3.1.1. Enumeration of Wastewater Isolates

**Figure 1:**
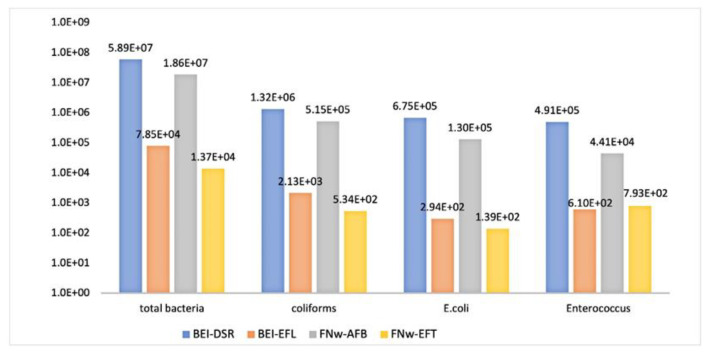
Microbiological analysis of 96 WWTP samples from the Beirolas-Lisboa and Faro Northwest-Algarve WWPTs of influent wastewater (BEI-DSR/FNw-ABF) and effluent wastewater (BEI-EFL/FNw-EFT), represented by the average numbers obtained for total aerobic bacteria, coliforms, *Escherichia coli,* and *Enterococcus* spp.

A total of 96 WWTP samples were obtained over the sampling period between November 2016 and January 2019, from influent and effluent wastewater at the Beirolas-Lisboa (BEI) and Faro Northwest-Algarve (FNw) WWPTs. The number of total aerobic bacteria and faecal contamination bacteria in the influent and effluent wastewaters were expressed by CFU/mL and are presented in Table S1. According to Figure 1, the microbiological analysis showed that the values of total aerobic bacteria in influent wastewaters of the BEI (BEI-DSR) and FNw (FNw-AFB) WWTPs were 5.89E+07 and 1.86E+07 CFU/mL, respectively. In the effluent wastewater of the BEI (BEI-EFL) and FNw (FNw-EFT) WWTPs, the treatment process produced a 3-log reduction relative to the influent of 7.85E+04 and 1.37E+04 CFU/mL, respectively. Additionally, in the other parameters analyzed (total coliforms, *E. coli*, *Enterococcus* spp.), there was a 3-log reduction at both WWTPs, with the exception of the number of *Enterococcus* spp. isolates in FNw-AFB and FNw-EFT WWTP, with a 2-log reduction of 4.41E+04 and 7.93E+02 CFU/mL, respectively.

#### 3.1.2. Enumeration of Antimicrobial-Resistant Gram-Negative Bacteria

The number of antibiotic resistant bacteria in the influent and effluent wastewaters was determined by plating wastewater samples on DLA culture medium, specifically for isolation of Gram-negative bacteria, supplemented with the antibiotics cefotaxime (CTX), gentamicin (GM), norfloxacin (NF), and trimethoprim-sulfamethoxazole (SXT). The DLA culture medium was used without antibiotics as a control for bacterial growth, and allowed us to determine the relationship between the number of total Gram-negative bacteria and the number of bacteria that grow with antibiotic selection.

**Figure 2:**
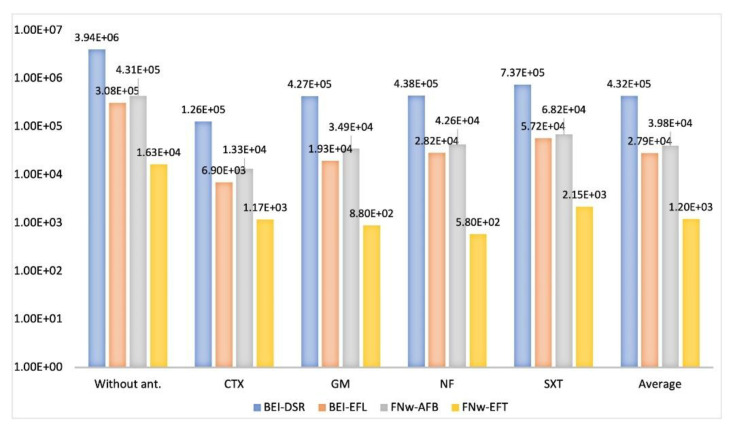
The number of colony-forming units that grew on Drigalsky without antibiotics (without ant.) and supplemented with the antibiotics: cefotaxime (CTX), gentamicin (GM), norfloxacin (NF), and trimethoprim-sulfamethoxazole (SXT). BEI-DSR: Beirolas-Lisboa influent wastewater; BEI-EFL: Beirolas-Lisboa effluent wastewater; FNw-AFB: Faro Northwest-Algarve influent wastewater; FNw-EFT: Faro Northwest-Algarve effluent wastewater.

According to Figure 2, when comparing the isolates grown on DLA culture medium without and with antibiotics, the CFU/mL for each antibiotic decreased of one log10 relative to the influent wastewaters of BEI-DSR and FNw-AFB. However, in the respective effluent wastewaters of these WWTPs, the CFU/mL decreased one or two log10 values depending on the antibiotic type. Considering the results for each antibiotic and the value of LRV (the logarithm of the ratio of pathogen concentration in the influent and effluent water), in BEI-WWTP there was a removal effectiveness of wastewater treatment of 99% (LRV of 2) of resistant isolates to the CTX antibiotic and 90% (LRV of 1) of resistant isolates to the GM, NF, SXT antibiotics. Additionally, in FNw-WWTP, removal efficiencies of wastewater treatment of 99% (LRV of 2) of resistant isolates to the GM and NF antibiotics and 90% (LRV of 1) of resistant isolates to the CTX and SXT antibiotics were achieved. Based on the results of 96 analyses, the average of the total number of resistant isolates was lower in the wastewater of Faro FNw-AFB (3.98E+04) and FNw-EFT (1.20E+03) than that of the Beirolas BEI-DSR (4.32E+05) and BEI-EFL (2.79E+04) WWPTs.

#### 3.1.3. Antimicrobial Susceptibility Profiles

**Figure 3:**
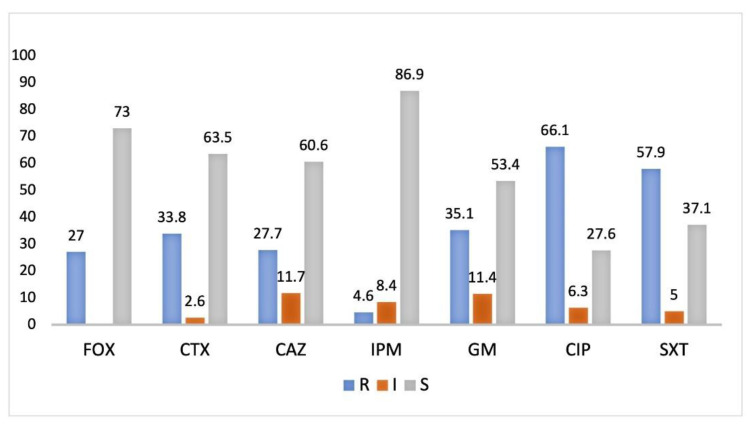
Antibiotic susceptibility frequency (%) of 315 isolates to cefoxitin (FOX), cefotaxime (CTX), ceftazidime (CAZ), imipenem (IPM), gentamicin (GM), ciprofloxacin (CIP), and trimethoprim-sulfamethoxazole (SXT), from influent and effluent wastewater from the Beirolas and Faro Northwest WWTPs. R-resistant; I-susceptible at increased exposure; S-susceptible at standard dose.

The antimicrobial susceptibility tests were performed for isolates that grew on DLA with antibiotics and according to colony morphologies. Yellow colonies (enterobacteria lactose positive) were preferentially selected for the antimicrobial susceptibility tests. The isolates were selected if they were mostly resistant to β-lactam and any of the other antibiotics. Subsequently, isolates with high susceptibility were not considered for molecular characterization, and a total of 315 isolates were selected. According to Figure 3, there was a deviation for the antibiotic ciprofloxacin (66.1%), followed by trimethoprim/sulfamethoxazole (57.9%), gentamicin (35.1%), and imipenem (4.6%). The resistance to the third generation cephalosporins cefotaxime (33.8%) and both ceftazidime and cefoxitin (20.0%; 27.7%) must be taken into account, because they are indicators of the production of enzymes (β-lactamases) that hydrolyze β-lactam antibiotics, and are strongly associated with bacteria isolated from hospitals and community-acquired infections.

**Figure 4:**
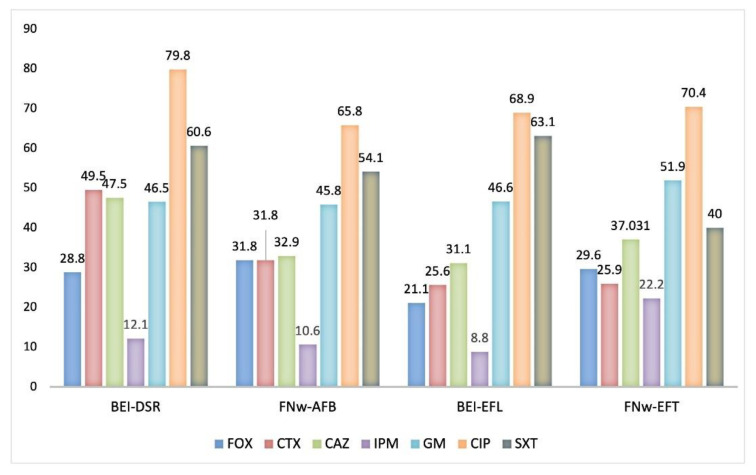
Percentage of resistant isolates of influent and effluent wastewater from the Beirolas and Faro Northwest WWTPs. Antibiotics tested: cefoxitin (FOX), cefotaxime (CTX), ceftazidime (CAZ), imipenem (IPM), gentamicin (GM), ciprofloxacin (CIP), and trimethoprim-sulfamethoxazole (SXT). BEI-DSR: Beirolas-Lisboa influent wastewater; BEI-EFL: Beirolas-Lisboa effluent wastewater; FNw-AFB: Faro Northwest-Algarve influent wastewater; FNw-EFT: Faro Northwest-Algarve effluent wastewater.

Considering the distribution of the WWTPs, the percentage of resistant isolates from influent and effluent wastewater of both the Beirolas (BEI-DSR; BEI-EFL) and Faro Northwest (FNw-AFB; FNw-EFT) WWTPs are presented in Figure 4. The lowest number of resistant isolates was found in the effluent wastewater, in accordance with the results obtained in the quantification of enterobacteria and other Gram-negative bacteria (Figure 2.). However, of relevance, the percentage of imipenem resistant bacteria isolated from the FNw-AFB (influent) and FNw-EFT (effluent) has increased from 10.6% to 22.2%.

### 3.2. Genomic Characterization

#### 3.2.1. Identification of Isolates by 16S rRNA

**Figure 5:**
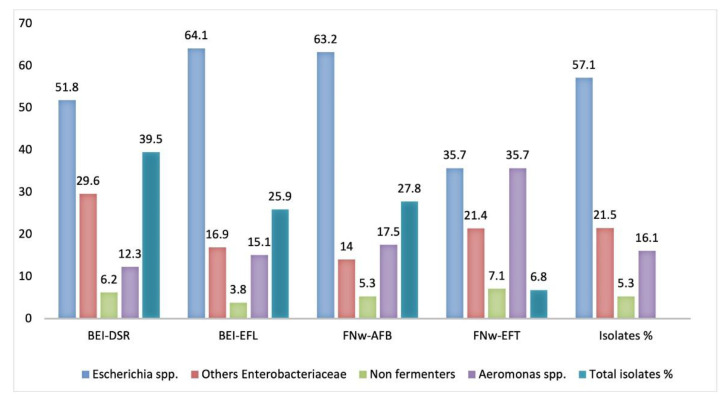
Frequency (%) of the 205 wastewater isolates by taxonomic identity: *Escherichia* spp., other *Enterobacteriaceae*, non-fermenter bacteria, and *Aeromonas* spp., from the Beirolas (BEI-DSR/EFL) and Faro (FNw-AFB/EFT) WWTPs. BEI-DSR: Beirolas-Lisboa influent wastewater; BEI-EFL: Beirolas-Lisboa effluent wastewater; FNw-AFB: Faro Northwest-Algarve influent wastewater; FNw-EFT: Faro Northwest-Algarve effluent wastewater.

Two hundred and five isolates (*n* = 205) resistant to two or more antibiotics, with emphasis on β-lactams, fluoroquinolones, and trimethoprim-sulfamethoxazole, were selected for identification and molecular characterization. After sequencing, the products of amplification obtained by 16S rRNA were successfully identified with similarity values higher than 98% with the type strain of an accurately named species, and were therefore considered members of that genus. Most of the wastewater’s 117 isolates (57.1%) were identified in genus *Escherichia*, namely *E. coli* (*n* = 95; 46.3%), *E. fergusoni* (*n* = 18; 8.7%) and *E. marmotae* (*n* = 4; 1.9%). Twenty-six isolates (12.7%) were identified as belonging to the genus *Klebsiella*, namely *K. pneumoniae* (*n* = 16; 7.8%), *K. michiganensis* (*n* = 7; 3.4%), *K. oxytoca*, *K. quasipneumoniae*, and *K. quasivariicola* (*n* = 1; 0.5% each). Nine different species of the genus *Aeromonas* (*n* = 33; 16.1%) were identified, namely: *A. caviae* (*n* = 16; 7.8%), *A. media* (*n* = 5; 2.4%), *A. hydrophila* (*n* = 3; 1.5%), *A. taiwanensis*, *A. veroni* and *A. dhakensis* (*n* = 2; 1.0% each) and *A. enteropelogenes*, *A. rivipollensis* and *A. sanarellii* (*n* = 1; 0.5% each). The remaining 29 isolates (14.1%) were distributed across 10 genera (Table S2). Relative to the distribution of the 205 isolates of each WWTP, and according to Figure 5, the wastewater isolates were grouped by taxonomic identity: *Escherichia* spp., other *Enterobacteriaceae*, non-fermenter bacteria, and *Aeromonas* spp.

The group *Escherichia* spp. was the majority in wastewater isolates of both WWTPs, contributing to 57.1% of the total of isolates studied. Although the influent from BEI-DSR had a lower number of *Escherichia* spp. isolates (51.8%), the number of isolates identified in this influent was higher (39.5%) than in the effluent BEI-EFL (25.9%), as well as from the influent FNw-AFB (27.8%) and effluent FNw-EFT (6.8%). It should be emphasized that *Aeromonas* spp. was found in 35.7% of isolates from effluent wastewater (FNw-EFT), and was higher than in effluent wastewater BEI-EFL, with 15.1% of isolates. At Faro WWTP, the last stage of effluent (FNw-EFT) was disinfection by ultraviolet radiation, which could explain the presence of *Aeromonas* spp. [27]. Among non-fermenter isolates, we should mention some species that were ubiquitous, not specifically pathogenic to humans, but possibly responsible for infections in the hospital and in the community environment, such as *Acinetobacter* spp. (*A. gerneri*, *A. pittii* and *A. nosocomialis*), *Comamonas* spp., *Ochromobactrum* spp. and *Uruburuella suis*, a novel species within the *Neisseriaceae* family.

#### 3.2.2. Detection and Sequencing of Antibiotic Resistance Genes

According to the susceptibility results, 205 wastewater isolates were selected for screening of resistance determinants, which was performed by PCR with specific primers, and after sequencing, 183 resistant genes were identified in 97 isolates, as presented in Table 1. The characterization of susceptibility to antibiotics and resistant genes found by isolate is presented in Table S3 (*n* = 97). Among the genes that expressed enzymes responsible for resistance to β-lactams, the gene *bla*_CTX-M-15_ was detected in 37 isolates, followed by *bla*_CTX-M-14_ (*n* = 8) and *bla*_CTX-M-27_ (*n* = 4), while the genes *bla*_CTX-M-1_, *bla*_CTX-M-2_, *bla*_CTX-M-32_, and *bla*_CTX-M-174_ were detected in one isolate each. The gene *bla*_SED-1_ was identified in *Citrobacter farmeri* and the gene *bla*_CTX-M-15_ in *Ochromobactum intermedium*. These genes express enzymes denominating the extended spectrum of β-lactamases (ESBLs), conferring resistance to third generation cephalosporins, and are prevalent among *Enterobacteriaceae*.

Additionally, in the β-lactamases family, carbapenemases that confer resistance to carbapenems can be found. In this study, the KPC-3 enzyme in one *K. oxytoca* with the *bla*_KPC-3_ gene was identified, which included a transposon *Tn4401* isoform b. Further, this bacterium co-produced a chromosomal β-lactamase OXY-2. This enzyme belongs to the group of narrow-spectrum β-lactamases, as well the OXA-2 produced by *Uruburuella suis*.

The resistance to ciprofloxacin (fluroquinolone) was promoted by the *aac(6′)-Ib-cr5* gene, which is a variant of the *aac(6′)-Ib* gene responsible for resistance to kanamycin, tobramycin, and amikacin. Twenty-six isolates carried *aac(6′)-Ib-cr5*, of which 17 isolates also harbored *bla*_CTX-M-15_. Co-carriage of aminoglycoside resistance genes (*aadA1*, *aadA2*, *aadA5*) and the trimethoprim resistance genes (*dfrA1*, *dfrA12*, *dfrA15*, *dfrA17*) were the most prevalent genes among our isolates. These genes are related to the class 1 integron detected in 49 resistant wastewater isolates. The gene cassette arrays *dfrA17–aadA5* were predominant (*n* = 20), followed by *dfrA1–aadA1* (*n* = 7) and *dfrA12–aadA2* (*n* = 9); 7 out of 9 isolates had a third gene cassette, *duf1010*, codifying a protein of unknown function.

#### 3.2.3. Molecular Typing Characterization

*E. coli* strains are mostly commensal, but the Enterobacterial species have become the most affected by ESBLs and pathogenic types. Therefore, to gain insight into the mechanism underlying this phenomenon, we assessed the diversity profiles and clonality within 26 *E. coli* isolates producing CTX-M-type. Regarding the phylogenetic groups, there was an equal distribution between group B2 (42.3%; 11/26) and group A (42.3%; 11/26); B1 (3/26) and D (1/26). The *pabB* gene is specific in isolates belonging to the serotype O25b, and after PCR amplification and sequencing seven (*n* = 7) *E. coli* O25b isolates (Table 2) were identified belonging to phylogroups B2 (*n* = 3) and A (*n* = 4), reported as pathogenic and commensal strains, respectively.

Using the MLST analysis, the sequence types (STs) found among the seven isolates belonged to serotype O25b:H4, which allowed us to verify a relationship between phylogroup B2 isolates with ST131, while in group A there was a diversity of STs.

#### 3.2.4. Whole-Genome Sequencing

The whole-genome sequencing of two *E. coli* 5301 (O25b:H4-B2-ST131) and *E. coli* 5281 (O25b:H4-A-ST410) was used to investigate the genomic content of antimicrobial resistance and virulence genes. The selection of these strains was in order to represent the pathogenic phylogenetic group (B2) and commensal (A) group from effluent (BEI-EFL10TM2) and influent (FNw-AFB7GM) wastewater from the Faro Northwest and Beirolas WWTPs, respectively. According to Table 3, *E. coli* 5301 had a greater number of genes, both considering resistance and virulence pathways, than *E. coli* 5281 from group A (commensal). Among the resistance genes, Class 2 integrons in *E. coli* 5281 were less common than Class 1 integrons in *E. coli* 5301, with the most prevalent *dfrA1–sat2–aadA1* array with gene cassettes encoding resistance to trimethoprim, streptothricin, and spectinomycin/streptomycin, respectively. Additionally, the *dfrA17*- *aadA5*-*qacEdelta1-sul1* array is the most prevalent among Class 1 integrons, with genes encoding resistance to trimethoprim, streptomycin, quaternary ammonium, and sulfonamide, respectively.

Multiple plasmids can be simultaneously detected within the same cell; both isolates had derivatives of IncF and IncH plasmids. The complete assembly of plasmids was not possible to obtain. Comparison of the virulence gene content of the two phylogenetic groups showed significant differences (Table 3). The group B2 isolate carried a higher number of virulence genes (19 genes) than the group A isolate (6 genes), and both had the same five virulence genes [Enterobactin; Aerobactin; CFA/1 (clade α); Type 1(clade γ1); Type IV].

## 4. Discussion

The quantification of the number of total aerobic bacteria and the number of resistant Gram-negative bacteria in this study allowed us to investigate the genomic features of antimicrobial resistance genes from influent and effluent wastewater from two WWTPs with different characteristics in Portugal.

The Beirolas (Lisboa) WWTP has the maximum treatment capacity of 54,500 m^3^/day, equivalent to a population of 213,510 inhabitants, while Faro Northwest (Algarve) WWTP has the maximum treatment capacity of 26.712 m^3^/day, equivalent to a population of 44,530 inhabitants. The difference in capacity between the two WWTPs may explain the lower number of total cultivable aerobic bacteria in Faro Northwest compared with Beirolas WWTP. However, in our study, resistant bacteria were found in effluents from both WWTPs. 

The quantification data on antimicrobial-resistant isolates from influent and effluent wastewater, and the proportion of antimicrobial resistant microorganisms, are based on selected isolates, usually involving the quantification of *E. coli* and therefore not representative of the entire population. In light of this data, we intended to cover a wide range of Gram-negative aerobic bacteria, specifically the most common etiologic agents associated with hospitals and/or community-acquired infections [2,28]. In fact, it should be emphasized that *Aeromonas* spp. was the dominant genus of resistant isolates from Faro Northwest WWTP. Additionally, the environmental isolates such as *Aeromonas* spp. and *Vibrio* spp. adopt different strategies to survive exposure to UV [27]. The high abundance of faecal bacteria in WWTP influent impact the bacteria number in the effluent, and consequently the efficiency of the treatment process. In our study, the microbial reduction after treatment produced an average of a 3-log reduction relative to influent values.

The effectiveness of wastewater treatment processes is measured using a concept called “log removal values” (LRVs) [13], which determine the removal effectiveness of wastewater treatment of 90% (LRV of 1) or 99% (LRV of 2) of resistant isolates. After the wastewater treatment, based on the activated sludge process, the effectiveness of treatment corresponds to a decrease of one or two log10. Similar results were obtained by Osińska 2017 [29], who studied the impact of the type of wastewater of four treatment processes. The process for activated sludge with the A2O system, similar to the two WWTPs in this study (Beirolas and Faro), was more effective in the removal of *E. coli* resistant to cefotaxime and doxycycline. However, although our results showed a high efficiency of reduction of the number of antibiotic-resistant bacteria during the treatment process, there continues to be discharge of these microorganisms into the natural environment. 

Considering the global priority of the World Health Organization [30] to address multi-resistant bacteria, those considered in our study were, in addition to *E. coli*, other *Enterobacteriaceae* family members, non-fermenter bacteria (*Acinetobacter baumannii* and *Pseudomonas aeruginosa*), and *Aeromonas* spp., producing mostly ESBLs and carbapenemases. Hospitals and healthcare facilities, where antibiotics are intensively used and where antibiotic-resistant bacteria may have a selective advantage over susceptible strains, are viewed as important hotspots for antibiotic-resistance genes [6]. Non-pathogen and pathogen species concentrations in wastewater are highly variable, making it practically impossible to measure for each and every individual pathogen. However, clinically relevant, resistant, and virulent isolates should be considered as a priority.

Identification of the bacteria at the species level was performed by 16S rRNA gene sequences; this has been by far the most common housekeeping genetic marker [31]. Indeed, the differentiation of species within the same genus is only possible by sequencing. For example, in our study, among the genus *Escherichia*, the species *E. coli*; *E. fergusoni* and *E. marmotae* were identified. For the genus *Aeromonas*, we identified nine species, mainly *A. caviae*, *A. media*, and *A. hydrophila*. The *Aeromonas* are widely distributed in the soil, aquatic environment, and in the water distribution system, due to forming biofilms in the water channels. The pathogenesis of *Aeromonas* species involves a series of virulence factors, and those associated with human infections are found in a wide variety of fresh vegetables, meat, and dairy products [32]. The three main pathogenic species of the genus are *A. hydrophila*, *A. caviae*, and *A. veronii biovar sobria*. *A. media* is an opportunistic pathogen for human and animals mainly found in aquatic habitats [33] and *A. dhakensis*, firstly isolated from children with diarrhea in Bangladesh in 2002, is widely distributed in the environment, and can cause a variety of infections in human and animals [34]. 

*Acinetobacter* species are ubiquitous in nature, and can be found in different environmental sources such as hydrocarbon contaminated areas, activated sludge [35] sewage, and dump sites, but also on vegetables, animals, and humans. For instance, *A. gerneri* was isolated from activated sludge [36]. *A. baumannii* has emerged as a clinically relevant pathogen [37]; however, the role of non-baumannii *Acinetobacter* in human infections is increasingly being reported, namely *A. pittii* in healthcare facilities in the United States of America [38] and China [39], and *A. nosocomialis* in Germany [40]. The genus *Comamonas* contains different species, and became clinically important after 1987, with various reports describing human infections such as cellulitis, peritonitis, endocarditis, meningitis, endophthalmitis, tenosynovitis, and pneumonia [41]. In contrast, *Uruburuella suis*, a novel species within the *Neisseriaceae* family, can be isolated from the lungs and hearts of different animals with lesions of pneumonia and pericarditis, respectively [42]. *Ochromobactrum intermedium* is an opportunistic pathogen especially found in hospital environments, and since 1999 seven cases have been reported due hospital-acquired infections [43], mainly related to immunocompromised patients with bacteremia, endophthalmitis, and pyogenic liver abscess [44].

The insufficient effect of the treatment process in removing bacteria harboring antibiotic resistance could be important for increased survival of specific multi-resistant clones. Despite the reduction in the total number of bacteria in the wastewater treatment process, also confirmed in our study, large numbers of bacteria exhibiting multi-drug-resistance and characterized by higher virulence could penetrate into the environment [5,28]. Effectively, the WWTPs are not specifically designed to remove antibiotic and antimicrobial resistant bacteria, and microbial contaminants in effluent wastewater are not usually subject to regulations or monitoring.

Taking into account the antibiotics used in clinical therapy, in this study we considered the antimicrobial resistance pattern to antibiotics from four classes: aminoglycosides and trimethoprim-sulfamethoxazole (resistant genes usually are in integrons), fluoroquinolones (resistant genes co-exist with ESBLs) and β-lactams (ceftazidime, cefotaxime, indicators of ESBLs), cefoxitin (indicators of AmpC β-lactamases), and Imipenem (indicator of class A, B and D carbapenemases group). The resistance to third generation cephalosporins cefotaxime and ceftazidime must be taken into account, as they are indicators of the production of enzymes (β-lactamases) that hydrolyse β-lactam antibiotics, and are strongly associated with multi-resistant bacteria from hospitals and community infections. Of relevance, 205 isolates found in this study were resistant to two or more antibiotics, including β-lactams, fluoroquinolones, and trimethoprim-sulfamethoxazole. 

The high content of microorganisms, the relative abundance of nutrients, and the presence of sub-threshold levels of antibiotic substances in wastewater [6] provide an environment favorable for survival, and subsequent transfer of genetic information between pathogenic and environmental microorganisms [6,30]. Among the resistance mechanisms, ESBL and carbapenemases were chosen as examples of clinically important mechanisms. These microorganisms can be regarded as a big “pool” of resistant genes, and the persistent entry and accumulation of antimicrobial agents in the environment facilitates their spread [29]. Herein, we report the identification of 183 resistance genes, including ESBL producers, of which CTX-M-15 was predominant. Moreover, of concern, this study describes for the first time an CTX-M-15 producing *Ochromobactrum intermedium* isolate and a SED-1 producing *Citrobacter farmeri*, an uncommon CTX-M-type extended-spectrum β-lactamase, only described in *Citrobacter sedlakii* to date [45], and that has also the ability to hydrolyze clinically extended-spectrum cephalosporins such as cefotaxime.

*Klebsiella pneumoniae* (Kp) is the causative agent of severe infections, including wound infections, bloodstream infections, meningitis, and pneumonia [28]. It is an increasingly resistant pathogen that has shown the ability to express diverse mechanisms, making the species multi-resistant to several clinical antimicrobial classes [3]. Ambler class B metallo-β-lactamases are a worldwide, increasingly distributed class, and represent a major treatment problem due to not being inhibited by any beta-lactamase inhibitor [4,9,14]. In Portugal, the Ambler class A KPC-3 is the most frequent carbapenemase described in healthcare-associated infections [2,3,28,46]. In this work, we describe a KPC-3 producing *K. oxytoca*, a rod-shaped bacterium that is closely related to *K. pneumoniae*, with *bla*_KPC-3_ included in a transposon *Tn4401* isoform b, mobile genetic element (MGE), which can favor horizontal dissemination capacity [28]. Future in-depth studies should be performed on the presence of carbapenem-resistant isolates in WWTPs.

*E. coli* are genetically diverse and predominantly harmless bacteria that are part of the normal gut flora of warm-blooded animals, including humans. *E. coli*, which cause extraintestinal infections (ExPEC), are known to carry several virulence and fitness genes that are not present in strains belonging to phylogroups A and B1 (commensal strains). ExPEC strains are associated with extra-intestinal infections like urinary tract infections, meningitis, pneumonia, and wound infections [47] and belong to group B2 or, to a lesser degree, to group D, whereas commensal isolates belong to groups A and B1. It is noteworthy that the *E. coli* 5301 of our study had different fimbrial types responsible for adherence and invasion. Offensive virulence factors such as the toxin CNF1 [48] and the secreted enterotoxin TieB encoded by the virulence gene *senB* play a role in the development of severe diarrhea in patients infected by enteroinvasive *E. coli* [49]; the *iss* gene promotes immune evasion by increasing serum survival [50]; and Hra1 is an outer membrane protein found in enteroaggregative *E. coli* [51]. Colicin is a bacteriocin produced by many *E. coli* in times of stress [52], as well as the uropathogenic-specific protein (USP), which acts as a genotoxin and is highly prevalent among uropathogenic and ulcerative colitis *E. coli* strains [53].

One key factor of this study involved the identification of the highly virulent clone O25b:H4-B2-ST131 ESBL-producing *E. coli*, which is thought to be responsible for the pandemic spread of the CTX-M-15 enzyme in hospital and community settings in Europe. The emergence of CTX-M-15-positive O25:H4 -B2-ST131 has been almost exclusively observed in the human population, despite it being previously reported in companion animals [54]. The importance of the identification of this clonal group in WWTPs can be inferred not only from its multi-resistance phenotype, but also from its extra-intestinal virulence capability.

## 5. Conclusions

In conclusion, the identification of the pandemic clone CTX-M-15-producing *E. coli* O25b:H4-B2-ST131 and the carbapenemase KPC-3-producing *K. oxytoca* in wastewater treatment plants is evidence of the risk of dissemination of clinically relevant antibiotic-resistant strains into the environment through treated wastewater. Additionally, to the best of the authors’ knowledge, our study reports the first detection of the ESBL CTX-M-15 producing *Ochromobactum intermedium* and the uncommon SED-1 in *Citrobacter farmeri.*

## Figures and Tables

**Table 1:** Distribution of 183 resistant genes identified in wastewater isolates from the Beirolas (BEI-DSR/EFL) and Faro (FNw-AFB/EFT) WWTPs (*n* = 97).

Gene Name	Gene Product	Antibiotic Resistance	BEI- DSR	BEI- EFL	FNw- AFB	FNw- EFT	Total Number of Resistance Genes
*aacA4*	Aminoglycoside 6’-N-acetyltransferase	kanamycin			1		1
*aadA1*	Streptomycin 3’-adenylyltransferase	streptomycin	5	3	1		9
*aadA2*	Streptomycin 3’-adenylyltransferase	streptomycin	6	2	3		11
*aadA5*	Streptomycin 3’-adenylyltransferase	streptomycin	9	7	3	3	22
*qnrB-1*	DNA-gyrase and topoisomerase	ciprofloxacin		2	1		3
*qnrB4*	DNA-gyrase and topoisomerase	ciprofloxacin		1	1		3
*qnrB13*	DNA-gyrase and topoisomerase	ciprofloxacin	1		1		2
*aac(6’)-Ib-cr5*	Aminoglycoside 6’-N-acetyltransferase	ciprofloxacin and kanamycin	19	4	2	1	26
*dfrA1*	Dihydrofolate reductase	trimethoprim	3	6	1		10
*dfrA7*	Dihydrofolate reductase	trimethoprim	3				3
*dfrA12*	Dihydrofolate reductase	trimethoprim	6	2	2		10
*dfrA15*	Dihydrofolate reductase	trimethoprim	2				2
*dfrA17*	Dihydrofolate reductase	trimethoprim	9	6	4	2	21
*dfrA27*	Dihydrofolate reductase	trimethoprim			1		1
*arr-3*	Rifampicin ADP-ribosyltransferase	rifamycin	1		1		2
*CTX-M-1*	Class A β-lactamase	cephalosporins	1				1
*CTX-M-2*	Class A β-lactamase	cephalosporins	1				1
*CTX-M-14*	Class A β-lactamase	cephalosporins	2	4	2		8
*CTX-M-15*	Class A β-lactamase	cephalosporins	22	6	6	3	37
*CTX-M-27*	Class A β-lactamase	cephalosporins	1		2	1	4
*CTX-M-32*	Class A β-lactamase	cephalosporins			1		1
*CTX-M-174*	Class A β-lactamase	cephalosporins	1				1
*OXY-2*	Class A β-lactamase	cephalosporins			1		1
*SED-1*	Class A β-lactamase	cephalosporins			1		1
*KPC-3*	Class A β-lactamase	carbapenem			1		1
*OXA-2*	Class A β-lactamase	oxacilin			1		1

Legend: BEI-DSR: Beirolas-Lisboa influent wastewater; BEI-EFL: Beirolas-Lisboa effluent wastewater; FNw-AFB: Faro Northwest-Algarve influent wastewater; FNw-EFT: Faro Northwest-Algarve effluent wastewater.

**Table 2:** Distribution of ESBL-producing *E. coli* wastewater isolates by multi-locus sequence typing (ST), serotype (O25b), and phylogroups B2 and A.

Isolates	Origin	ESBL	ST	Serotype	Phylogroup
5301	BEI-EFL_10TM	CTX-M-15	131	O25b	B2
5302	BEI-EFL_12CTX	CTX-M-15	131	O25b	B2
5262	BEI-DSR_6TM	CTX-M-174	131	O25b	B2
5318	FNw-EFT_11GM	CTX-M-15	44	O25b	A
5281	FNw-AFB_7GM	CTX-M-15	410	O25b	A
5064	FNw-AFB_10CTX	CTX-M-15	10	O25b	A
5295	BEI-EFL_9TM	CTX-M-14	23	O25b	A

Legend: BEI-EFL: Beirolas-Lisboa effluent wastewater; BEI-DSR: Beirolas-Lisboa influent wastewater; FNw-EFT: Faro Northwest-Algarve effluent; wastewater; FNw-AFB: Faro Northwest-Algarve influent wastewater. The number is indicative of the number of water samples and the letters indicative of the antibiotic used. TM: Trimethoprim; CTX: cefotaxime and GM: gentamicin. ESBL: extended spectrum β-lactamase; ST: sequence type.

**Table 3:** Genetic features of *E. coli* 5301 pathogenic isolate (B2) compared with *E. coli* 5281 commensal isolate (A), from effluent (BEI-EFL10TM2) and influent (FNw-AFB7GM) wastewater.

Isolates	Resistance Genes		Plasmids	Virulence Genes		
	β-Lactamases	Others Genes	Replicon	Offensive Factors	Iron Uptake	Fimbrial Types
5301	CTX-M-15; OXA-1; EC5	*dfrA17*; *aadA5*; *qacEdelta1*; *sul1*; *aac(3)-IIa*; *catB3*; *aac(6’)-Ib-cr5*;	FIA; FIB; FII; I1-I	CNF1; Iss; Hra1; Colicin Ia; Usp; senB	Enterobactin; Aerobactin; Yersinobactin; ChuA; sitA	CFA/1 (clade α); Type1(clade γ1); Yad (clade γ4); Yeh (clade γ4); Ybg (clade π); Yfc (clade π); P (clade π); Type IV
5281	CTX-M-15; TEM-1	*dfrA1*; *sat2*; *aadA1*	HI1A; HI1B; R		Enterobactin; Aerobactin	CFA/1 (clade α); Type1(clade γ1); Sfm (clade γ1); Type IV

Legend: CNF1-cytotoxic necrotizing factor; Iss-increased serum survival lipoprotein; hra-heat-resistant agglutinin; USP-uropathogenic specific protein; senB-enterotoxin.

## Data Availability

Data sharing not applicable.

## Supplementary Materials

The following are available online at https://www.mdpi.com/2076-2607/9/3/576/s1; Table S1: List of *E. coli* isolates and their susceptibility patterns; Table S2: Distribution the bacterial species identified by 16S rRNA gene sequencing by influent and effluent wastewater of Beirolas (BEI-DSR and BEI-EFL) and Faro Noroeste (FNw-AFB and FNw- EFT) WWTPs; Table S3: Distribution of selected isolates from the influent and effluent wastewater treatment plants Beirolas-Lisboa and Faro Northwest-Algarve by susceptibility to antibiotics and resistant genes.
