# Supplementary material for: An Update on Wastewater Multi-Resistant Bacteria: Identification of Clinical Pathogens Such as Escherichia coli O25b:H4-B2-ST131-Producing CTX-M-15 ESBL and KPC-3 Carbapenemase-Producing Klebsiella oxytoca
Source: Microorganisms. 2021 Mar 11;9(3):576. doi: 10.3390/microorganisms9030576 (PMC8001128; doi:10.3390/microorganisms9030576)
Supplement: Supplementary file 1 [file microorganisms-09-00576-s001.zip › microorganisms-1117797-sup/Table S1.pdf]

BEI-DSR					BEI-EFL				
	total bacteria	coliforms	*E.coli*	*Enterococcus*		total bacteria	coliforms	*E.coli*	*Enterococcus*
21/11/16	2.20E+08	1.16E+06	2.40E+05	1.70E+04	21/11/16	6.40E+05	5.40E+02	8.00E+01	9.00E+01
12/12/16	8.80E+07	3.00E+06	1.28E+06	9.00E+04	12/12/16	2.68E+05	3.00E+03	3.00E+02	3.00E+02
03/01/17	6.40E+07	1.21E+06	2.00E+05	1.04E+06	03/01/17	2.00E+04	1.50E+03	1.60E+02	1.32E+02
06/02/17	2.34E+07	4.00E+04	1.00E+04	6.50E+04	06/02/17	1.40E+05	2.00E+03	6.50E+02	2.00E+03
06/03/17	1.90E+07	3.00E+04	1.70E+03	9.10E+04	06/03/17	6.00E+04	4.60E+02	8.00E+01	5.80E+01
11/04/17	6.50E+07	1.00E+04	2.00E+02	4.80E+03	11/04/17	4.50E+04	3.00E+03	1.70E+02	2.40E+02
08/05/17	1.46E+08	4.80E+04	2.00E+06	2.00E+06	08/05/17	5.50E+04	3.00E+02	2.40E+02	2.16E+03
05/06/17	3.20E+07	4.80E+04	2.00E+06	2.00E+06	05/06/17	4.50E+04	2.00E+02	2.00E+02	1.24E+02
03/07/17	3.80E+08	1.35E+06	4.00E+05	4.30E+05	03/07/17	8.90E+04	6.20E+02	6.00E+02	5.00E+02
11/09/17	1.08E+08	1.02E+07	1.20E+06	1.02E+06	11/09/17	1.00E+05	1.50E+03		1.30E+02
10/10/17	7.00E+07	1.08E+05	4.50E+04	1.31E+05	10/10/17	5.20E+04	2.60E+02	1.00E+01	1.40E+02
12/12/17	2.80E+06	2.70E+04	2.00E+03		12/12/17	4.80E+04	3.80E+03	1.00E+03	3.00E+01
09/01/18	9.10E+05	3.00E+03			09/01/18	4.60E+03	3.20E+02		
05/02/18	7.40E+06	2.20E+05	1.50E+04	1.00E+04	05/02/18	2.40E+03	2.00E+01	1.00E+01	1.00E+01
05/03/18	3.20E+05	6.00E+04	1.00E+04		05/03/18	6.80E+02	4.50E+02		
02/04/18	2.12E+06	8.00E+03	2.00E+03		02/04/18	6.00E+02	4.00E+01	2.00E+01	
07/05/18	1.80E+06	1.00E+04			07/05/18	1.40E+03	1.40E+02	1.00E+01	
04/06/18	7.50E+06	1.06E+06	1.50E+05	3.10E+05	04/06/18	3.70E+03	9.40E+02	5.00E+01	1.10E+02
02/07/18	1.02E+07	1.51E+06	1.81E+05	1.77E+05	02/07/18	7.00E+03	4.70E+03	4.40E+02	2.10E+02
11/09/18	5.80E+07	6.20E+05	1.60E+05	1.13E+05	11/09/18	1.40E+03	9.00E+01	6.00E+01	4.40E+03
02/10/18	4.60E+07	6.10E+05	1.16E+05	4.50E+03	02/10/18	4.00E+03	1.00E+02	7.00E+01	7.70E+02
06/11/18	2.10E+07	2.30E+05	1.40E+05	1.10E+05	06/11/18	1.20E+05	2.30E+04	3.00E+02	2.60E+02
03/12/18	1.50E+07	4.60E+06	3.50E+06	3.00E+05	05/12/18	4.50E+04	1.28E+03	2.30E+02	1.10E+02
07/01/19	2.50E+07	5.50E+06	3.20E+06	1.41E+06	07/01/19	1.31E+05	2.80E+03	1.50E+03	4.30E+02
	5.89E+07	1.32E+06	6.75E+05	4.91E+05		7.85E+04	2.13E+03	2.94E+02	6.10E+02

FNw-AFB					FNw-EFT				
	total bacteria	coliforms	*E.coli*	*Enterococcus*		total bacteria	coliforms	*E.coli*	*Enterococcus*
23/11/16	1.60E+07	3.60E+04	1.10E+04	2.00E+02	23/11/16	2.00E+03	8.00E+01		
14/12/16	1.83E+07	3.00E+05	1.06E+05	5.10E+03	14/12/16	1.48E+04	2.40E+01		
04/01/17	1.46E+07	2.80E+05	7.80E+04	3.20E+03	04/01/17	1.50E+04			
08/02/17	5.50E+07	3.00E+05	9.40E+04	3.00E+02	08/02/17	3.80E+04	7.90E+01		
08/03/17	4.80E+06	3.00E+05	5.20E+04	1.72E+04	08/03/17	9.00E+03	1.56E+02		
13/04/17	1.80E+07	3.00E+05	2.40E+05	1.00E+04	13/04/17	2.80E+04	1.17E+02		
10/05/17	2.16E+07	3.00E+04	3.00E+04		10/05/17	1.80E+04	4.00E+03		
07/06/17	4.10E+07	2.00E+06		2.80E+05	07/06/17	4.40E+04			
05/07/17	1.20E+06	3.00E+04			05/07/17	2.20E+03	1.00E+02		
13/09/17	1.40E+07	1.35E+05	4.00E+04	4.30E+04	13/09/17	5.30E+03	2.20E+02		
12/10/17	3.00E+06	2.80E+05	1.47E+05	3.90E+04	13/10/17	1.80E+02	2.30E+02	1.00E+01	
15/12/17	9.00E+05	4.50E+04	4.00E+03		15/12/17	6.20E+03	1.00E+03	1.00E+01	2.20E+02
11/01/18	3.90E+05	1.10E+04			11/01/18	2.20E+04	3.00E+01		
07/02/18	3.00E+06	7.00E+04	4.20E+04	1.00E+04	07/02/18	9.00E+02	3.00E+01		
07/03/18	3.00E+06	4.50E+04	1.50E+04	7.00E+03	07/03/18	9.00E+02	3.00E+01		
04/04/18	3.00E+06	7.00E+04	4.20E+04	1.00E+04	04/04/18	2.70E+03	1.10E+02	2.00E+01	
09/05/18	1.43E+06	3.60E+04	1.30E+04	8.00E+03	09/05/18	2.70E+03	1.00E+02		
06/06/18	5.20E+07	5.50E+06	9.60E+05	1.00E+05	06/06/18	7.60E+03	1.60E+02		
04/07/18	3.40E+06	9.40E+05	1.70E+05	4.00E+04	04/07/18	5.70E+03			4.20E+02
13/09/18	7.20E+07	2.90E+05	1.40E+05	6.60E+04	11/09/18	7.00E+03	4.20E+02	9.00E+01	1.80E+03
04/10/18	6.00E+07	5.00E+05	3.00E+05	7.00E+04	04/10/18	1.50E+04	4.00E+02	1.10E+02	2.80E+03
08/11/18	2.40E+07	4.60E+05	8.00E+04	5.00E+04	08/11/18	1.70E+03	2.10E+03	3.00E+02	6.00E+01
05/12/18	7.50E+06	1.00E+05	7.00E+04	4.30E+04	05/12/18	3.00E+04			1.00E+01
09/01/19	9.20E+06	3.00E+05	1.00E+05	8.00E+04	09/01/19	5.00E+04	1.30E+03	4.30E+02	2.40E+02
	1.86E+07	5.15E+05	1.30E+05	4.41E+04		1.37E+04	5.34E+02	1.39E+02	7.93E+02
